# Bone-conduction hearing in elephants and humans: a middle-ear comparative study

**DOI:** 10.3389/fauot.2026.1744613

**Published:** 2026-07-14

**Authors:** Caitlin E. O’Connell-Rodwell, Jodie L. Berezin, Anbuselvan Dharmarajan, Alessio Pignatelli, Rachel Chen, Francisco Uzal, Xiying Guan, Sunil Puria

**Affiliations:** 1 Eaton-Peabody Laboratories, Massachusetts Eye and Ear, Boston, MA, United States; 2 Department of Otolaryngology, Head & Neck Surgery, Harvard Medical School, Boston, MA, United States; 3 Department of Biostatistics, Johns Hopkins University, Baltimore, MD, United States; 4 Harvard Medical School, Boston, MA, United States; 5 School of Veterinary Medicine, University of California, Davis, Davis, CA, United States; 6 Communication Sciences and Disorders, Wayne State University, Detroit, MI, United States; 7 Graduate Program in Speech and Hearing and Biosciences and Technologies, Harvard Medical School, Boston, MA, United States

**Keywords:** 3D laser Doppler vibrometry, air conduction, bone conduction, cochlear fluid inertia, ear canal, elephant hearing, human hearing, middle ear

## Abstract

The elephant’s middle ear (ME) boasts ossicles approximately nine times more massive than humans’, with eardrums seven times larger. These anatomical features contribute to enhanced low-frequency sensitivity to air-conducted (AC) sound, but their contribution to bone-conduction (BC) hearing is less understood. To characterize BC hearing in elephants, we employed 3D laser Doppler vibrometry to measure ossicular motion in cadaveric temporal bones from both African and Asian elephants, as well as humans in response to BC stimulation. We analyzed velocity measurements projected along the piston direction at the umbo, stapes, and cochlear promontory across a frequency range of 12–11,000 Hz. Differential velocity (V_diff_) was calculated as the difference in the velocity of the piston (V_pist_) and the velocity of the promontory (Vprom) divided by the velocity of the promontory [(V_pist_-V_prom_)/V_prom_]. V_diff_ plateaued above the BC resonant frequency–1.2 kHz for humans, reflective of prior studies, and 400 Hz for elephants. Below their respective resonant frequencies, the stapes V_diff_ magnitude for elephants was three to four times greater than that of humans, and both decreased at approximately 12 dB/octave until about 200 Hz. Surprisingly, both elephant and humans exhibited a plateau in their stapes V_diff_ below 200 Hz, rather than continuing to decrease at the 12 dB/oct rate. This suggests that both species have better low-frequency sensitivity for bone conduction than previously predicted. These results indicate that the larger ossicular and eardrum mass distributions play a crucial role in lowering BC resonance and enhancing low-frequency sensitivity. Elephants may further enhance BC hearing below about 200 Hz by contracting a skeletal muscle that occludes the ear canal, achieving a similar result to the occlusion effect in humans. These mechanisms may explain elephants’ ability to detect ground-borne acoustic signals over long distances.

## Introduction

Asian (*Elephas maximus*) and African elephants (*Loxodonta africana*), the largest terrestrial mammals, communicate over long distances using vocalizations with a fundamental frequency between 10 and 20 Hz at a sound pressure level of 120 dB (Poole, 1999; Stoeger and Baotic, 2016; Baotic and Stoeger, 2017; Wierucka et al., 2021). Like in humans, the middle ear influences the elephant’s ability to hear (Hemilä et al., 1995; Reuter et al., 1998; Homma et al., 2010).

The terrestrial middle ear can take a variety of configurations, ranging from the “microtype” form found in small rodents, associated with high-frequency hearing, in which the malleus is attached directly to the bony walls of the ME cavity, to the “freely mobile” form in larger mammals, associated with lower-frequency hearing (Fleischer, 1978). Within this range, there is a great diversity of eardrum and ossicular shapes and sizes (Doran, 1877; Nummela, 1995). While ossicular heterogeneity has been recognized, the relationship between their configuration and frequency sensitivity remains elusive.

Asian and African elephants are valuable species to help better understand these relationships because they possess the largest middle ears of all extant terrestrial vertebrates (Hemilä et al., 1995; Nummela, 1995) and the lowest frequency range of hearing (Heffner and Heffner, 1982). Ossicles of both elephant species are approximately nine times more massive than those of humans, and the elephant ME has an order of magnitude better sensitivity at lower frequencies in response to AC sound (O’Connell-Rodwell et al., 2024a). Given that these elephant species share a similar structure, mass, and magnitude of difference compared to the human ME, we will refer to both species collectively as “the elephant.”

While the eardrum and ossicles transmit acoustic signals to the cochlea, an alternate mechanism of sound transmission to the cochlea is through skull vibrations, known as hearing by bone conduction (BC). Five factors contribute to BC hearing: ear canal sound radiation, inertia due to the ME bones, inertia due to cochlear fluids, cochlear wall compression, and cerebrospinal fluid pressure transmission. The relative contribution of the first three factors for BC hearing in humans is believed to be most significant (Stenfelt and Goode, 2005). However, the relative contributions of these factors in other species, such as elephants, remains unknown.

Investigating the relationship between structure and function at the boundary of anatomical diversity provides critical insights into hearing by BC. Notably, an enlarged malleus has been linked to enhance BC sensitivity in various species, including small and large ones, through the ME inertia mechanism (Hemilä et al., 1995; Mason and Narins, 2002; Mason, 2006). In this context, understanding the mechanics of BC hearing in elephants, whose ossicular morphology is highly specialized, could offer valuable insights.

In a related previous study, Stenfelt et al. (2002) experimentally increased ossicular mass by adding mass to the umbo and stapes in human cadaveric temporal bones. They measured vibration velocity at the umbo, stapes, and promontory during BC stimulation and calculated V_diff_. They found that tripling the malleus mass at the umbo reduced the middle ear resonant frequency by more than an octave. Importantly, this umbo mass led to up to a 10-fold increase in stapes and umbo V_diff_ magnitudes below the middle ear resonant frequency.

Measurements of BC hearing in elephants, therefore, offer a natural experiment to assess how ossicular mass influences sound transmission through the middle ear during BC stimulation. Such data could greatly enhance our understanding of the biomechanical principles governing low-frequency hearing in large mammals. Here, we collected 3D ossicular motion and report the projected motions along the piston direction for the first time. Our quantitative analysis of 3D ossicular motion and sound transmission in response to BC sound stimulation in elephant temporal bones extends down to 12 Hz. For comparison, we conducted similar experiments in human temporal bones. In our previous report, we reported velocities in three directions from the ossicles of two elephant ears and one human ear (O’Connell-Rodwell et al., 2019). This study significantly expands that preliminary report.

## Methods

All methodology presented here, aside from certain bone conduction procedures, have been described in detail previously (Gottlieb et al., 2016, 2018; O’Connell-Rodwell et al., 2024a) and are presented briefly below.

### Specimen preparation

Elephant measurements were collected from three temporal bones: one from an African elephant and two from Asian elephants. All specimens were donated post-mortem from zoo and sanctuary facilities. Each temporal bone was from a different animal: the African elephant was labeled ETB2 (right ear, 4-month-old female) and the Asian elephants were labeled ETB3 (left ear, died before birth and harvested *in-utero* male), and ETB6 (right ear, 72-year-old female). Variability in measurements was observed (Supplementary Figure S2); however, we could not draw any conclusions about whether this variation was due to age, sex, or species due to the limited sample size.

Human measurements were collected from three temporal bones, harvested from the Massachusetts General Hospital morgue (Mass Eye & Ear IRB #2021P002348). Human specimens were labeled as TB18 (left ear, sex and age not available), TB19 (left ear, 46-year-old male), and TB20 (left ear, 58-year-old female) and were used for vibrometry. Human specimens were obtained from donors with no history of otologic disease, and all elephant and human temporal bones were visually screened for middle-ear pathologies prior to experimentation.

Both elephant and human temporal bones were cut down to fit within a bowl-shaped temporal bone specimen holder. The middle-ear cavity was accessed through the posterior-inferior facial recess using an otologic drill, and any remaining fluid drained. The ear canal lengths were approximately 20–40 mm for elephants (based on the ability to trim down the specimen) and about 10 mm long for humans. The time between death, temporal bone dissection, and start of measurements is not well determined. But it is likely that it varied between multiple weeks to months for the elephants. For humans it was likely less.

All specimens were stored in saline with a few drops of Betadine (Povidone-Iodine) as an antibacterial and kept in the refrigerator between measurement sessions. During the day of the experiment, it was kept at room temperature. When not being used for experiments, specimens were wrapped in food-grade plastic wrap and aluminum foil and stored in a −20 °C freezer.

Each specimen underwent varying experimentation time windows, due to sample preparation differences between the smaller and larger samples, as well as the difficulty of accessing the targets on larger specimens. As such, we aimed to keep freeze-thaw cycles to a minimum, not more than three, while refrigerating between experiments if they had to be repeated on subsequent days.

### Experimental setup

**FIGURE 1:**
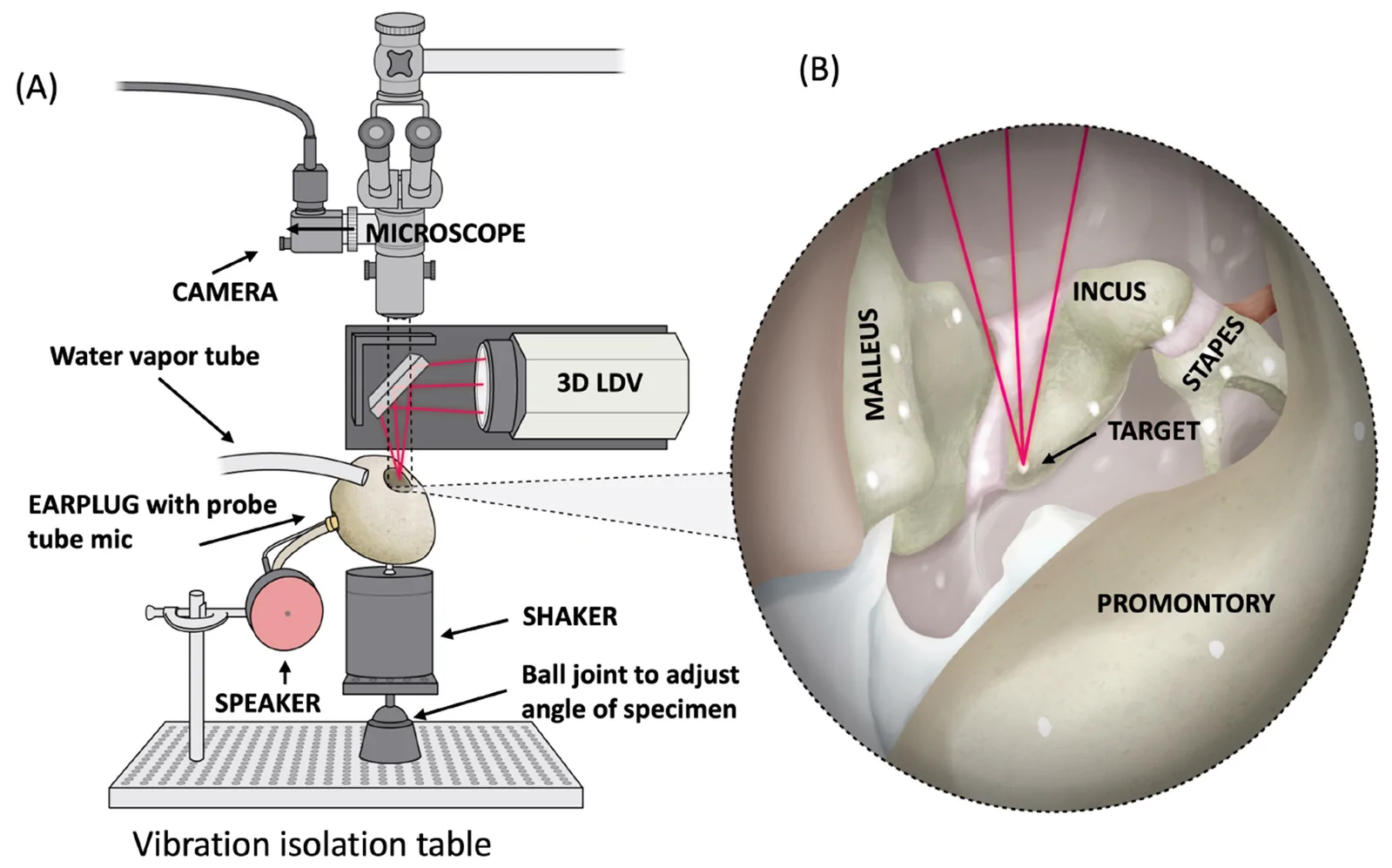
Experimental set-up for bone conduction velocity measurements. **(A)** Experiments were performed on a vibration isolation table, where the temporal bone was mounted to a shaker that provided vibration stimulation. A 3D LDV was mounted on a motorized stage with a dichroic mirror, with specimen viewed through a microscope, allowing visualization of the specimen. **(B)** Cutaway of an elephant temporal bone with the three LDV laser beams aligned to a single point on reflective tape placed on the incus. The promontory is also displayed with target locations. Upon vibration delivery, velocity measurements of the ossicles and promontory were recorded. The water vapor tube was attached to a humidifier that kept the specimen moist during the measurement session. The sound source (Speaker) was not used during BC measurements (Modified from O’Connell-Rodwell et al., 2024a).

A 25–30 mm-long threaded rod (size #10–32) was affixed to the inferior bony portion of each temporal bone, while the other end was screwed into a mini-shaker (4810, Brüel+Kjær, Naerum, Denmark) used to stimulate the temporal bone with mechanical vibrations (Figure 1A). The mini-shaker was mounted on a rotation-tilt ball joint, which enabled the adjustment of the temporal bone’s orientation such that the 3D LDV and camera could access the middle-ear cavity. The mini-shaker and ball joint were then attached to a breadboard mounted on a vibration isolation table (Nexus, T46H, Thorlabs, Newton, NJ) to reduce vibrational intrusions.

A soft-foam ear plug containing a sound source and probe-tube microphone was inserted into the ear canal for ear canal pressure measurements. The ear canal was sealed, which may have created a “closed acoustic tube” enhancing bone conduction (Stenfelt et al., 2003) and impacted the velocity measurements reported here.

Four reflective tape targets were placed along each ossicle in relatively the same position amongst specimens. Three targets were placed along the cochlear promontory as a measure of the input reference for bone conduction velocity measurements (Figure 1B). The velocity of each target was measured using a motorized 3D LDV controlled by SyncAv software. Additional details for the 3D ossicular motion measurement methodology have been described previously (O’Connell-Rodwell et al., 2024a).

Velocity data from the umbo, incus (point closest to the incudostapedial joint), and stapes head were measured for both species. Sample numbers for data presented hereafter are as follows:

- ETB2: umbo (*n* = 2), incus (*n* = 1), stapes (*n* = 1), promontory (prom) (*n* = 1)
- ETB3: incus (*n* = 1), stapes (*n* = 2), prom (*n* = 2)
- ETB6: stapes (*n* = 3), prom (*n* = 2)
- TB18: stapes (*n* = 2), prom (*n* = 2)
- TB19: incus (*n* = 1), stapes (*n* = 2), prom (*n* = 4)
- TB20: umbo (*n* = 3), incus (*n* = 3), stapes (*n* = 6), prom (*n* = 4).

The n’s here indicate test-retest measurements made on different days which were averaged to produce a single measurement for each specimen. Given the constraints of facial-recess opening of the specimen and access with the three laser beams of the 3D-LDV, we could not measure the umbo and stapes within the same sessions for four of the specimen and so only the stapes was measured in all six. However, incus velocity was measured in four of the six specimen which are reported in Supplementary Figure S2.

### Source stimulus

The mini-shaker delivered BC stimulation consisting of pure tones generated in an 8192-sample buffer sampled at 30 kHz. For elephant specimens, a series of 55 logarithmically spaced pure tones from 7 Hz to 13 kHz was used. For human specimens, 48 logarithmically spaced pure tones from 17 Hz to 11 kHz were used. The noise floor was measured by repeating measurements with an output voltage close to 0 volts. The shaker used produced constant acceleration for constant voltage, and thus the velocity decreased generally at about −20 dB/decade. The individual X, Y, Z umbo, stapes and promontory velocities were typically 10 mm/s in the 20–40 Hz range, decreasing linearly at higher frequencies. These velocities were less than the levels used in Stenfelt et al. (2002). The corresponding pressures in the occluded ear canal during BC were typically less than 100 dB SPL. In our companion AC stimulation study, the ear canal pressures were typically less than 100 dB SPL. All of these levels are well below non-linear range of 120 dB SPL.

The noise floor varied but the SNR of the individual velocity components stayed typically 30–40 dB above the noise floor. Above about 7–8 kHz, the SNR was lower and closer to 10 dB for some of the ears measured. Velocity data was processed using the SyncAv Toolbox, consisting of custom scripts in MATLAB (MathWorks Inc., 2023).

### Piston direction projections

We determined the direction of piston motion by projecting its three-dimensional motions onto one-dimensional axes aligned with relevant anatomical directions. For the stapes and incus, this direction is perpendicular to the plane of the footplate, while for the umbo, it is perpendicular to the plane of the tympanic ring. Similarly, the cochlear promontory motion was projected perpendicular to the plane of the stapes. Coordinate transformations were computed by aligning the original LDV reference frame with the desired anatomical reference frame. Consequently, the reported measurements are one-dimensional velocities that have been transformed onto their anatomically relevant directions. Differential piston-direction velocities (V_diff_) were then computed by normalizing each measurement by the cochlear promontory (V_prom_), as depicted in the formula: $V_{diff}=\frac{V_{pist}-V_{prom}}{V_{prom}}$ . Thus, V_diff_ indicated the difference in vibration velocity of the umbo or ossicle compared to that of the cochlear promontory. Promontory velocity magnitudes were found to be comparable among both elephant and human specimens.

### BC ME resonance

**FIGURE 2:**
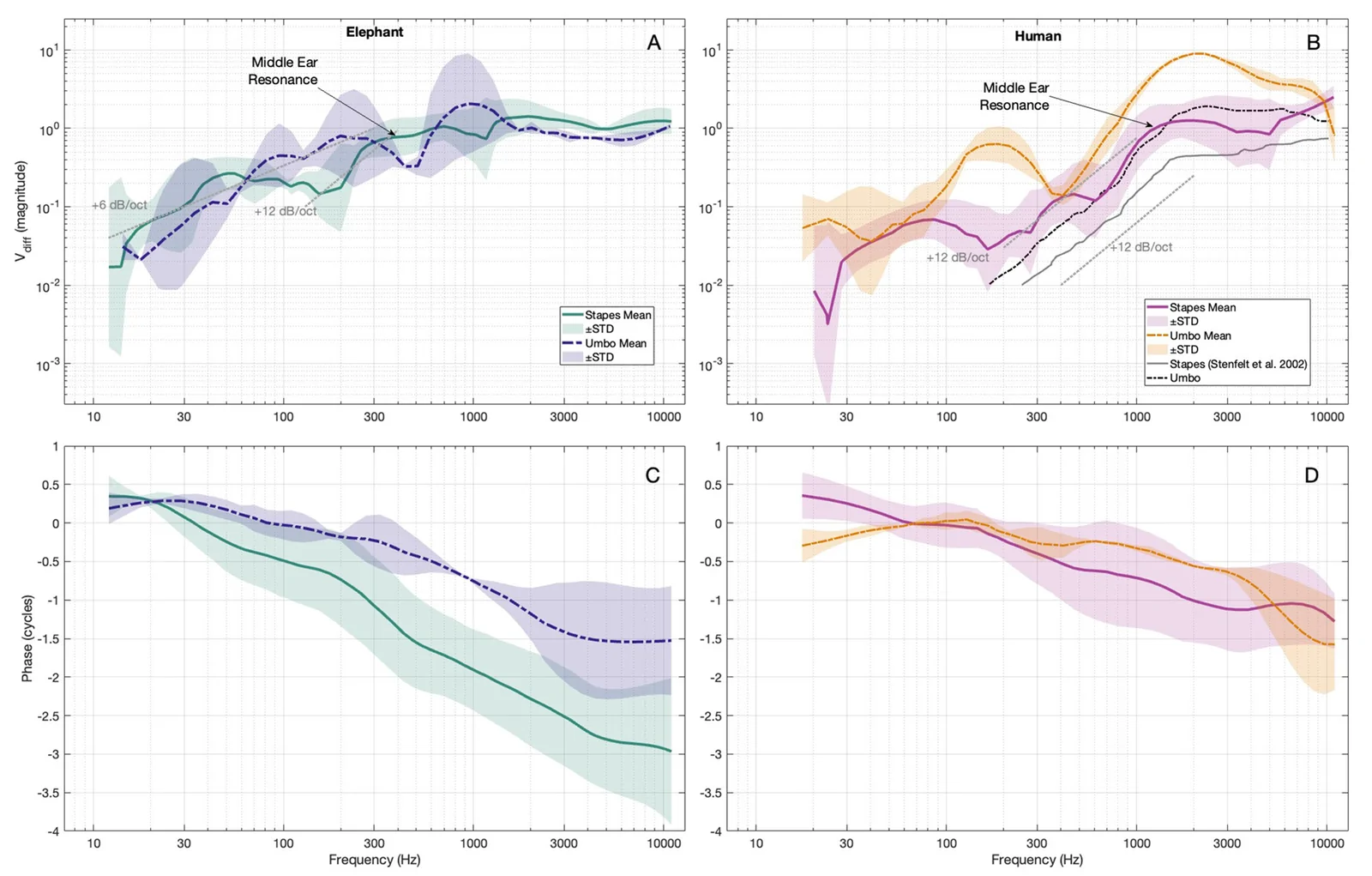
Bone conducted V_diff_ at the umbo and stapes mean and STD for Elephant **(A, C)** and Human **(B, D)**. Multiple measurements in individual ears were averaged across different days. Velocities were normalized by the promontory velocity [(Vpiston–Vprom)/Vprom]. Number of temporal bones: elephant umbo (*n* = 1), elephant stapes (*n* = 3), human umbo (*n* = 1), human stapes (*n* = 3). Number of total measurements per ossicle per species (elephant umbo = 2, stapes = 6; human umbo = 3, stapes = 10). Those individual ears were then averaged. Lines show mean and shaded areas show ±1 STD. For reference, Stenfelt et al. (2002) measurements of stapes and umbo V_diff_ magnitude are shown in **(B)**.

With BC stimulation, the magnitude of V_diff_ at the stapes generally increased with frequency and then nearly flattened out (Figure 2B). The frequency at which the flattening began is estimated to be the ME resonant frequency for BC, which is slightly higher than the resonant frequency measured with AC stimulation (Homma et al., 2009). It was estimated to be near 1.2 kHz for human and this resonant frequency was approximately close to previous findings (Stenfelt et al., 2002; Homma et al., 2009). Using the same approach, we determined that the elephant BC resonance frequency was approximately 400 Hz, which was higher than our previous estimate for AC ME resonance of 300 Hz.

### Unwrapping phase corrections

In the initial design of the experiments, we chose frequency spacing based on anticipated phase shifts in humans to achieve continuous phase changes without unwrapping errors. However, the unexpected larger phase decrease in elephants led to 2π unwrapping errors. To rectify this, we manually corrected the errors to ensure a smooth transition in the phase.

### Group delay calculations

To interpret phase accumulation and examine the effects of flexibility of middle-ear substructures, including the bending of ossicles and slippage across joints (Puria and Allen, 1998), we calculated the group delay (GD). A straight-line was fit (using a least-squares method) to the phase on a linear-frequency axis ranging from an octave below each animal’s respective ME resonant frequency to three octaves above it. GD was calculated as the rate of change of the phase (slope), multiplied by −1. Smaller group delays indicate stiffer structures, whereas larger group delays indicate greater flexibility.

### Statistical analysis

We assessed whether normalized V_diff_ magnitudes and phases differed significantly between elephants and humans across the range of frequencies measured. The logarithm of the average velocity for each specimen was used for the statistical analysis (Supplementary Figure S2). Because the umbo was difficult to access while simultaneously measuring the stapes, we had an inadequate number of measurements for umbo V_diff_, so those were not statistically analyzed.

Separate models were created for the low and high frequency ranges. Low frequency was defined as 17–400 Hz, and high frequency as 1.2–11 kHz. These frequency ranges were selected based on visual inspection of Figures 2C, D to allow for direct comparisons between elephant and human. Human relative velocities exhibited an increasing magnitude in the 17–1,200 Hz range and a plateau in the 1,200–11,000 Hz range, while elephant relative velocities showed an increasing magnitude in the 11–400 Hz range and a plateau in the 400–13,000 Hz range. For each frequency range, log magnitude and phase were separately modeled. For each, a linear fixed-effect model and a linear mixed-effects models were performed using the “lm” function in base R and the “lmer” function in the R package lmerTest (Kuznetsova et al., 2017), respectively.

All models included the logarithm of frequency (log was chosen because measurements were log-spaced and visual inspection of Figures 2C, D suggested linearity in the log-scale) and species (elephant vs. human) as fixed-effects predictors. Interaction between species and log frequency was included when it significantly improved models based on nested-model ANOVA. When the interaction term was included, the lower limit of the log frequency range was subtracted from the log frequency predictor, so that the coefficient for species could be interpreted as the mean difference between elephant and human at the lower limit of the range (17 Hz and 1.2 kHz for low and high frequency models, respectively). Mixed-effect models additionally included a random-effect term of specimen to account for repeated measures. Model assumptions of linearity, homoscedasticity, and normality were visually assessed and met for all models (see Code for details).

Based on descriptive analysis, we suspected the relationship between log-magnitude of stapes velocities and log-frequency was approximately piece-wise linear with higher slopes at lower frequencies and a plateau at higher frequencies in both species. To test whether the plateau occurred at significantly different frequencies in the two species, we fitted a piecewise linear model where the knot location could vary (as well as the slopes) by species. The model was fitted using R using the Levenberg-Marquardt algorithm for non-linear models from the “minpack.lm” library. Bootstrap was used to calculate confidence intervals for the difference in knot locations.

Statistical analyses were performed using RStudio (Posit Team, 2023) and R (R Core Team, 2023, ver 4.3.1). All models were assessed at *α* = 0.05 for significance.

## Results

### Umbo and stapes piston-direction motion from BC stimulation

Figure 2 reports mean and STD of V_diff_ measured at the umbo and stapes for elephant (left column) and human (right column). The top panel shows the magnitude of V_diff_ (in dimension-less units), while the bottom row shows the phase (in cycles).

Elephant V_diff_ magnitudes at the umbo and stapes were similar but exhibited some differences (Figure 2A). Both reached a magnitude plateau of ~1 above the ME resonance frequency of about 400 Hz. Below the ME resonance, the umbo V_diff_ decreased with a slope of about 6 dB/oct (Figure 2A). The stapes V_diff_ magnitude decreased with a slope of 12 dB/oct from the ME resonance until about 200 Hz. Between 50 and 200 Hz, it plateaued with minimal fluctuation. At frequencies lower than 50 Hz, the magnitude of stapes V_diff_ decreased further.

Human stapes V_diff_ reached a plateau in magnitude of ~1 above the ME resonance frequency of about 1.2 kHz (Figure 2B). Between the ME resonance and 200 Hz, stapes V_diff_ magnitude decreased with a slope of about 12 dB/oct, and reached a plateau below 150 Hz. Although human V_diff_ magnitudes for umbo were quite variable due to the low number of specimens measured, there was a trend toward higher magnitudes than for stapes V_diff_ (Figure 2B). Our data exhibit the same trends as Stenfelt et al. (2002), which are that it rapidly increases with frequency, reaches a peak near the ME resonance frequency, and then appears to plateau. While present umbo V_diff_ magnitude was higher, these measurements fall within the range of previously reported data (Stenfelt et al., 2002). The piecewise linear model confirmed that humans stapes V_diff_ magnitude reached a plateau at a frequency significantly higher than elephants [approximately 2.73 times higher, bootstrap 95% CI = (1.70, 3.62)]. One limitation of this analysis is that some of the transition frequencies were influenced by the smoothing applied (see Supplementary Figure S1 for data corresponding to Figure 2, before smoothing).

Both the elephant umbo and stapes average phases were flat in 10–20 Hz range, then decreased to −1.5 cycles at about 4 kHz for the umbo and −3 cycles for the stapes at 10 kHz (Figure 2C). The elephant umbo phase had a shallower slope than the stapes phase and crossed over 0 cycles at approximately 100 Hz, while the stapes had a steeper slope and crossed 0 cycles near 30 Hz.

The human umbo and stapes V_diff_ phases were similar from about 50–300 Hz, both remaining somewhat flat and crossing over 0 cycles at about 70 Hz (Figure 2B). After about 300 Hz, the umbo phase had a less steep slope than stapes V_diff_ did at the same frequencies, but they crossed again at about 5 kHz. Both phases stayed above approximately −1.5 cycles.

**FIGURE 3:**
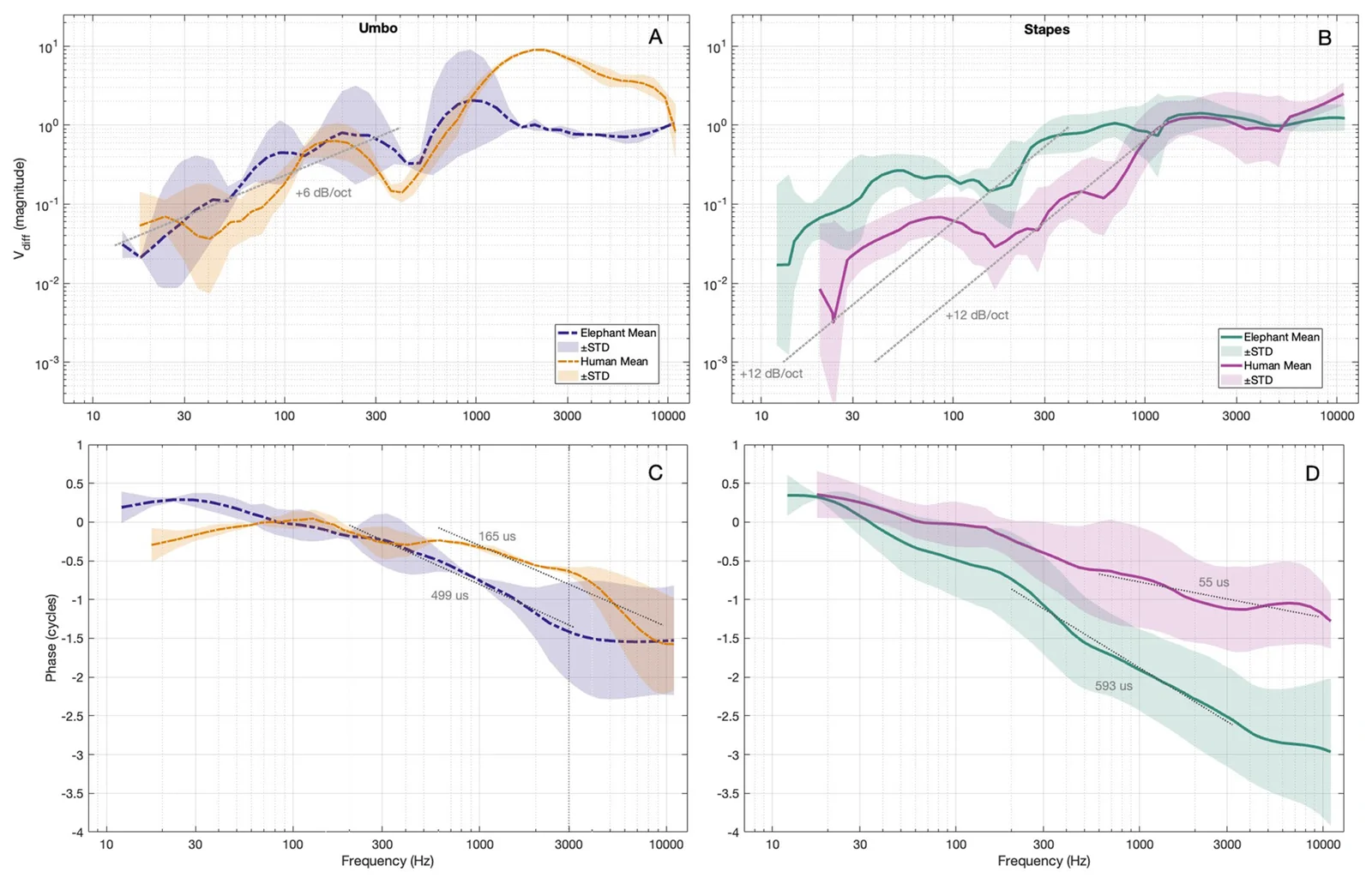
Umbo **(A, C)** and stapes **(B, D)** elephant and human V_diff_ with BC simulation. The data presented here are the same as Figure 2, but organized by measurement location for direct comparisons between elephant and human. The group delays (dashed gray line) were calculated as straight line fits to the linear phase around their respective ME resonant frequencies.

For ease of comparison across species, Figure 3 contains the same data as presented in Figure 2. Figures 3A, C directly compare the elephant and human umbo, and Figures 3B, D directly compare the elephant and human stapes V_diff_.

Overall, the average magnitude of the elephant umbo V_diff_ was relatively similar to human until about 1 kHz, and at higher frequencies the human umbo velocity is greater than elephant by up to +18 dB (Figure 3A). The elephant V_diff_ umbo phase was more positive than human from about 20–100 Hz, similar to human from 100 to 500 Hz, and then more negative for frequencies between 1 and 8 kHz (Figure 3C).

The elephant stapes V_diff_ was overall considerably different from that of human, as demonstrated statistically in Table 1. At low frequency, magnitude significantly increased as frequency increased. Elephants have significantly higher magnitude (*e*^1.634^ ≈ 5.1 times greater, p = 0.038), but the rate of change of magnitude with frequency does not differ across the two. Phase significantly decreased as frequency increased. Near approximately 17 Hz, phase did not differ significantly between elephants and humans. However, as frequency increased, phase decreased more substantially in elephants relative to humans (*p* < 0.001).

At high frequency, magnitude did not change substantially with frequency and did not differ much across species. Phase decreased slightly in humans as frequency increased. At 1.2 kHz, elephants had lower phase than humans, and as frequency increased, phase decreased more steeply than in humans (*p* < 0.001). Visually, V_diff_ magnitude of the stapes in elephant was higher than human between 20 and 600 Hz by a factor of about two-to-three times and then similar thereafter (Figure 3B). However, in both species, the slopes for stapes V_diff_ magnitudes below their respective ME resonance frequencies decreased by 12 dB/oct until about 200 Hz. Below 200 Hz, they both reached a plateau at first, and then decreased further for frequencies below about 50 Hz. The average V_diff_ stapes phase varied from approximately an octave below the ME resonance frequency to three octaves above it, with the steepest variations occurring in this range (Figures 3C, D). Furthermore, the rate of change was generally greater for elephant than human.

### Middle-ear group delays

We quantified the phase changes around ME resonance frequencies by fitting a straight-line to the phase data and calculating the group delay. These results are shown for the umbo in Figure 2C and for the stapes in Figure 2D. The group delay for elephant stapes was the largest (593 μs). The fact that it was greater than at the umbo (499 μs) suggests that BC transmission propagated from the umbo to the stapes, like in air conduction. Whereas the group delay for human at the umbo (165 μs) was greater than at the stapes (55 μs), suggesting that transmission propagated from stapes toward the umbo. Stenfelt et al. did not report V_diff_ phase, but instead they reported the phase of the ratios V_st_/V_prom_ and V_u_/V_prom_ for human. Both phases were approximately 0 cycles below 1.2 kHz, the ME resonant frequency. At higher frequencies, the phases decreased and V_st_/V_prom_ decreased to about −0.25 cycles, and for V_umbo_/V_prom_ to about −1.1 cycles both near 10 kHz (see Supplementary Figure S1D). This also suggests that transmission propagated in reverse from the stapes toward the umbo during human BC stimulation. As discussed in Discussion, we argue that this reverse transmission propagation is initiated by fluid inertia in the cochlea, when it is fluid filled.

## Discussion

In a previous study, we reported measurements comparing sound-driven (air-conduction) ossicular motion in elephants and humans (O’Connell-Rodwell et al., 2024a). In this complementary study, we report bone-conduction stimulation on the same specimens measured during the same sessions. Among the five distinct BC mechanisms, we primarily focus on the ossicular inertia and cochlear fluid inertia mechanisms, while briefly discussing the ear canal sound radiation mechanism.

Below the elephant BC resonant frequency, we showed that elephants had three-to-four times higher V_diff_ stapes magnitude than humans. Both decreased with a slope of 12 dB/oct until about 200 Hz, after which the slope was not as steep for lower frequencies, which was unexpected (Figure 3B). We argue below that this was likely due to the closed ear canals in our preparations. Above the human and elephant BC middle ear resonant frequencies, the stapes BC magnitudes in human and elephant were similarly close to approximately 1.

The phase of both human and elephant stapes with BC was about +¼-cycle near 20 Hz. As frequencies of the BC stimulus increased, both species’ stapes phase decreased, the elephant reaching about −3 cycles near 10 kHz, while human only reached about −1.1 cycles (Figure 3D). From these phase measurements, we calculated group delays around their resonant frequencies and found that elephant had nearly 10 times more group delay at the stapes than did human.

### Middle ear resonant frequency during BC stimulation

In human, we found an average ME resonance of about 1 kHz for AC (O’Connell-Rodwell et al., 2024a) and 1.2 kHz for BC stimulation. These results fall within the range previously reported (Homma et al., 2009). We expected the BC resonant frequency in elephant to be lower than in human, given that the ear drum of the elephant is seven times larger, the malleus 11 times heavier, and incudes masses eight times heavier, with an overall ossicular mass approximately nine times higher than that of humans’ (Nummela, 1995; O’Connell-Rodwell et al., 2024a). Assuming equal stiffness, this anatomy suggests that elephants would have a resonant frequency about three times lower than humans for BC, as was true of AC, where we found the AC resonant frequency for elephant to be around 300 Hz (O’Connell-Rodwell et al., 2024a). Our results match our expectations, as we found a BC resonance frequency of about 400 Hz for elephant (Figure 2A).

For elephant, we showed the stapes has five-to-ten times greater sensitivity to air conducted sounds than humans below the middle ear resonance (O’Connell-Rodwell et al., 2024a), most likely due to the combination of greater ossicle and eardrum mass with larger eardrum area. In human mass-loading experiments, Stenfelt et al. (2002) showed that below the ME resonant frequency, the magnitude of the umbo velocity in response to BC stimulation increased by a factor 20 due to a 100 mg increase in mass at the umbo (approximately a factor of four). Further, they found that the added mass on the umbo reduced the resonance frequency and increased the relative stapes velocity V_diff_ at the footplate, suggesting that the ossicles do influence BC hearing, particularly below the ME resonance frequency. Thus, it is likely that the three- to-four times higher stapes velocity V_diff_ magnitude for elephant, below its ME resonant frequency, was due to their ossicle masses. As a side note, Stenfelt et al. (2002) added 20 mg to the stapes mass, which increased V_diff_ at the footplate up to 10 dB and indicates higher cochlear input.

While we used a 3D LDV and projected the motion along the piston directions, Stenfelt et al. (2002) used a 1D LDV measured along the piston direction through the ear canal and a perforation in the eardrum. In comparison to Stenfelt’s mean umbo and stapes V_diff_ magnitudes, our results for human are generally higher (Figure 2B), but our means fall within the range reported in Stenfelt et al. (2002) and follow similar trends. One advantage of the present 3D LDV method is that measurements could be made through the facial recess while leaving the ear canal intact and closed. This stands in contrast to Stenfelt’s measurements, which necessitated an open ear canal and a hole in the eardrum for BC measurements. However, facial recess opening also necessitated a mastoidectomy, which could have potentially affected our BC measurements, though the extent of this impact remains unknown.

### ME group delay and direction of ossicle motion

The umbo and stapes phases were generally steeper around the ME resonant frequencies. Consequently, we calculated the group delays from an octave below and three octaves above the ME resonance frequencies. The elephant group delays are the largest BC delays reported for any species (Figures 3C, D), which is consistent with delays measured for AC transmission in elephants.

Temporal bone vibrations produced by BC stimulation exert inertial forces on the ossicular chain, eardrum, and incompressible cochlear fluids. Since the round window membrane is significantly more compliant than the stapes footplate, there is a difference in the volume velocities of the two windows due to BC stimulation, which produces a pressure difference across the two windows and sets up the classic traveling wave due to BC (v. Békésy, 1932; Stenfelt, 2007; Kim et al., 2011; Puria and Rosowski, 2012). Thus, when cochlear fluid is present, their inertial forces partially set the stapes in motion during BC stimulation, which propagates laterally toward the umbo. For humans, the average BC group delay at the umbo of 165 μs was three times more than 55 μs at the stapes. This indicates that the stapes footplate initiated ossicular motion, consistent with the cochlear fluid inertia stimulation. This can also be seen in some individual ears where the phase at the incus was also greater than at the stapes (TB19, TB20 in Supplementary Figure S2).

For elephants, the group delay was 499 μs at the umbo and 593 μs at the stapes. This means that the delay at the stapes lagged behind the delay at the umbo by a factor of 1.2 times. This finding is opposite of what we and Stenfelt et al. (2002) measured in humans. It suggests that elephant BC ossicular vibration was initiated at the umbo and then propagated toward the stapes in a manner similar to AC. We do not know what the state of the cochlea was in these specimens. However, one interpretation for this surprising result is that the elephant inner ears were drained of cochlear fluids during our experiments, due to repeated freezing and thawing over the course of specimen preparation and measurements (Ravicz et al., 2000). If true, the cochlear inertial component would be absent, leaving only the relative motion between the eardrum and ossicle inertial components to vibrate due to the acceleration of the middle ear cavity walls. This can be seen in an individual elephant ear where the phase of the incus and stapes was generally greater than that of the umbo (ETB2, Supplementary Figure S2).

What implications might a drained cochlea have for measurements of elephant BC? Stenfelt et al. (2002) conducted BC measurements of the stapes before and after draining the human cochlea and Guan (2025) simulated this effect in a computational model. Their findings indicate that after draining the cochlea, V_diff_ magnitude at the stapes decreased slightly below the ME resonance and increased slightly above it. Consequently, it is probable that our measurements of the drained elephant cochlea might underestimate V_diff_ magnitude to some extent. The effect of draining the cochlea on the phase of V_diff_ was less phase (Guan, 2025). Thus, with an intact elephant cochlea, one might expect even greater phase and thus group delay in V_diff_.

### Ossicular group delay during bone conduction as a novel indicator of cochlear fluid status

Temporal bone experiments are important for understanding the mechanics of hearing in species such as humans and elephants, where *in vivo* experiments are not feasible. As demonstrated by the series of experiments reported here, a reliable method for determining whether the cochlea was fully or partially drained would have been valuable. The realization that the inertia of the inner ear fluid during BC causes mechanical vibration to propagate primarily from the stapes toward the umbo—the reverse of the air conduction (AC) direction—offers a novel basis for detecting cochlear fluid status in each experimental preparation.

It is generally assumed that the contents of the cochlea are incompressible, so the volume flow at the oval and round windows should be equal and occur at opposite phases. Previously, several approaches have been reported to determine whether the cochlea was drained or partially drained. One approach is to measure the velocities of the stapes and round window membrane (RWM) and look for a 180° phase difference between them under AC stimulation (Kringlebotn, 1995; Stenfelt et al., 2004b). This approach is complicated by the fact that the RWM exhibits multiple vibrational modes above a few 100 Hz, potentially obscuring the expected phase relationship and rendering results unreliable at higher frequencies (Stenfelt et al., 2004b).

A refinement of this approach is to measure velocity at multiple points on both the stapes and the RWM and calculate their respective volume velocities, thereby averaging over the higher-order vibrational modes of the RWM (Stenfelt et al., 2004a). This method was validated for AC stimulation; however, for BC stimulation, minor perilymph leaks prevented a direct comparison between intact and drained cochlear conditions. Moreover, even for AC stimulation, this technique requires a large number of measurement points, resulting in considerable experimental time.

Yet another, more mechanically disruptive approach is to measure the ear canal pressure-to-cochlear pressure gain under AC stimulation (Puria et al., 1997). It was demonstrated that air bubbles introduced into the scala vestibuli and scala tympani detectably altered this transfer function (Puria et al., 1997). However, this approach is not suitable for BC experiments, as the surgical access required to place an intracochlear pressure sensor would significantly perturb the cochlear fluid inertial mechanics under investigation.

Of all the previously reported methods for determining cochlear fluid status, the approach of measuring phase along the ossicular chain and calculating the group delay is perhaps the most direct, and is particularly well-suited for BC experiments. Critically, this method exploits the fact that the presence of cochlear fluid is a prerequisite for the reversed propagation direction characteristic of BC stimulation: without fluid, the inertial mechanism that drives motion from the stapes toward the umbo is absent, and the characteristic phase progression along the ossicular chain is expected to be altered or reversed.

### Ear canal contributions to BC hearing

BC stimulation at the lowest frequencies measured might be more sensitive than previously reported. Below their ME resonant frequencies, V_diff_ magnitude for both human and elephant stapes decreased at 12 dB/oct and reached a plateau below about 200 Hz, rather than continuing to decrease at 12 dB/oct as expected (Figure 3B, dashed lines). In both cases, the ear canal was closed with a foam ear plug, which could have resulted in a pressure build up. In the Stenfelt et al. (2002) setup, a 25 mm long open tube glued against the bony ear canal served as an artificial ear canal. However, because it was open, it would not necessarily have enabled pressure to build up at these low frequencies. Contributing to this effect was the perforation made in the eardrum to access the middle ear bones.

Further measurements in human temporal bones revealed that for frequencies below approximately 700 Hz, the majority of the low-frequency bone conduction sound is generated by vibrations in the ear canal, which are then transmitted to the stapes as AC stimulation (Stenfelt et al., 2003; Stenfelt and Goode, 2005). Indeed, it is widely known that when an individual plugs their ear canal using their finger, with a sound-attenuating foam plug, or with an audio earbud, they encounter the occlusion effect. This effect causes them to perceive their own voice as louder compared to when the ear canal is unobstructed. Elephants cannot plug their ear canal, but they are known to have a skeletal muscle that contracts and occludes the opening of the ear canal (O’Connell-Rodwell et al., 2019). This contraction would create a “closed acoustic ear canal” similar to our experimental setup that could enhance their bone conduction. Thus, a possible explanation for increased sensitivity in BC below 200 Hz is that V_diff_ at the stapes was dominated by vibration borne ear canal pressure rather than ossicular motion, or cochlea fluid inertia.

We can speculate that if V_diff_ was measured with an open ear canal, its magnitude would continue to decrease at 12 dB/oct and at 20 Hz (where elephants vocalize) reach about 2.2·10^−3^ , while measured V_diff_ at the stapes was 6.7 · 10^−2^ (Figure 3B) corresponding to a ratio of approximately 30, or 30 dB. This suggests that elephants can, in principle, improve their BC sensitivity by at least 30 dB by simply contracting the muscle surrounding the external auditory meatus. Similarly, we can extrapolate from the human stapes V_diff_ and obtain a ratio of about 8.4, or 18 dB at 100 Hz (near the low-end of the fundamental pitch for humans). Thus, near the low-end of the human speech range, a 20 dB boost is conceivable at some frequencies, simply by plugging our ear canals. These are estimates from our experimental setup and the exact amounts will differ depending on the volume of the ear canal that is occluded (Stenfelt et al., 2003; Stenfelt and Reinfeldt, 2007).

### Implications of low-frequency AC and BC hearing for elephants

All elephant species produce low-frequency vocalizations that they detect (Heffner and Heffner, 1982) and respond to (Poole et al., 1988; Langbauer et al., 1989; Poole, 1999; McComb et al., 2003; Soltis et al., 2014; Stoeger and Baotic, 2017; O’Connell-Rodwell et al., 2022) for long-distance communication up to and, under certain conditions, greater than 4 km away (Langbauer et al., 1991; McComb et al., 2003). Elephants are able to produce vocalizations with a fundamental frequency of 10–20 Hz (Poole et al., 1988; Stoeger and Baotic, 2016; Baotic and Stoeger, 2017; Wierucka et al., 2021; O’Connell-Rodwell et al., 2024b), which are generated at such high amplitudes that they propagate along the surface of the earth as Rayleigh waves (O’Connell-Rodwell et al., 2000).

Seismic and airborne signals often travel at different velocities, which produces a time of arrival cue that increases at greater distances (O’Connell-Rodwell et al., 2000; Arnason et al., 2002). As such, these seismic signals provide a supplement to air-borne sounds which are more limited by environmental conditions (i.e., atmospheric and substrate) and frequency-dependent absorption (Marten and Marler, 1977; Wiley and Richards, 1978, reviewed in O’Connell-Rodwell, 2007). Both female and male African savanna elephants maintain home ranges with sizes of approximately 41 to up to 9,700 km^2^, with an average of about 1,900 km^2^ for females and 2,700 km^2^ for males (Benitez et al., 2022). Elephants use this long-distance communication to maintain contact with extended family members (Poole et al., 1988; McComb et al., 2003), advertise reproductive status (Poole et al., 1988; Langbauer et al., 1991; Poole, 1999), and relay information about the environment or potential danger (Nair et al., 2009; Soltis et al., 2014; Sharma et al., 2020) across long distances.

Given the elephant’s long-distance acoustic communication needs, a low-frequency middle-ear resonance and a skeletal muscle that can purposefully occlude the ear canal represent two critical anatomical advantages that would facilitate elephant sensitivity to low frequency BC hearing across extended distances.

## Conclusions

In this study, we report on BC sensitivity of the middle-ear in elephant and human, complementing a previous study reporting on AC sensitivity using the same specimens (O’Connell-Rodwell et al., 2024a). We present evidence suggesting that the elephant’s ME is adapted for low frequency hearing, capable of processing both AC and BC sounds. This adaptation is facilitated by a large eardrum, that generates larger forces to overcome the expected loss of hearing due to the large ossicles (Hemilä et al., 1995; Nummela, 1995). Field research has found that elephants communicate over long distances (Langbauer et al., 1991; Larom et al., 1997; McComb et al., 2003; Baotic et al., 2018), and our findings have implications for understanding how elephants might communicate over even longer distances through ground vibrations without the same constraints as airborne sound transmission (O’Connell-Rodwell et al., 2000). Previously, BC data in human was only reported to 100 Hz (Stenfelt et al., 2002). Our measurements include stimuli as low as 12 Hz for a few of the elephant specimens and 17 Hz for human. Surprisingly, the BC magnitude for both human and elephant tended not to continue decreasing at lower frequencies. Rather, they both plateaued between 50-200 Hz, then decreased again at lower frequencies. We suspect that these results are due to ear canal occlusion within our experiment. Based on this, we propose that selectively closing the ear canal, using voluntary muscles at the opening of the ear canal, might boost sensitivity of BC hearing by nearly 30 dB, achieving similar results to the occlusion effect in humans. One caveat is that the inner ears of the elephant specimens might have been drained, which would likely result in an underestimation of the effects reported in this study.

Little is currently known about BC hearing of non-human animals. However, these results provide the first insight into BC hearing of elephants, a mammal with hearing abilities that are quite similar to those of humans but with some important differences. Our findings demonstrate improved BC stimulation to the cochlea at low frequencies, which aligns with what is known of long-distance, low-frequency communication in elephants. However, this is the first physiological evidence suggesting that detection could also occur through bone conduction. This study also provides new context to understanding human BC hearing and the contributions of ME mass and ear canal acoustics to BC hearing in general.

## Supplementary Material

Supplementary Information

The Supplementary Material for this article can be found online at: https://www.frontiersin.org/articles/10.3389/fauot.2026.1744613/full#supplementary-material

## Figures and Tables

**TABLE 1:** Results of linear and linear mixed effects models.

Frequency range	Outcome	Predictor	Linear model				Linear mixed-model with specimen random intercept			
			Coefficient	SE	t statistic	P	Coefficient	SE	t statistic	P
Low frequency (17–400 Hz) N = 6, n = 133	Log (stapes magnitude)	Log (frequency)	0.501	0.092	5.435	<0.001*	0.498	0.076	6.569	<0.001*
		Species (elephant vs. human)	1.617	0.161	10.037	<0.001*	1.634	0.537	3.042	0.038*
	Stapes phase	Log (frequency)	−0.264	0.049	−5.373	<0.001*	−0.264	0.030	−8.856	<0.001*
		Species (elephant vs. human)	−0.033	0.127	−0.256	0.798	−0.077	0.282	−0.274	0.796
		Log(frequency)*species (elephant vs. human)	−0.209	0.068	−3.077	0.003^*^	−0.200	0.041	−4.862	<0.001*
High Frequency (1.2–11 kHz) N = 6, n = 100	Log (Stapes magnitude)	Log (frequency)	0.086	0.105	0.817	0.416	0.084	0.093	0.904	0.368
		Species (elephant vs. human)	−0.092	0.135	−0.685	0.495	−0.083	0.335	−0.247	0.817
	Stapes phase	Log (frequency)	−0.125	0.112	−1.121	0.265	−0.125	0.041	−3.036	<0.003*
		Species (elephant vs. human)	−1.126	0.210	−5.355	<0.001*	−1.168	0.479	−2.437	0.069
		Log (frequency)*species (elephant vs. human)	−0.339	0.161	−2.109	0.038*	−0.326	0.059	−5.497	<0.001*

Velocity data was extracted from Supplementary Figure S2. N = number of specimen included in the model, n = number of total measurements included in the model. All models included an intercept (not shown in table), the natural logarithm of frequency and species (with human as reference) as predictor. The interaction term between log (frequency) and species is included only when significant based on a nested-model ANOVA. When the interaction term is included, the lower limit of the log (frequency) range is subtracted from the log (frequency) predictor, so that the coefficient for species can be interpreted as the mean difference between elephant and human at the lower limit of the range (17 Hz and 1.2 kHz for low and high frequency models, respectively). Mixed models included a random intercept for each specimen, in addition to the fixed effect predictors already included in linear models.

An asterisks (*) represents significance at α = 0.05.

## Data Availability

The original contributions presented in the study are available from harvarddataverse https://doi.org/10.7910/DVN/FZBZDM and further inquiries can be directed to the corresponding authors.
