# Supplementary material for: Bone-conduction hearing in elephants and humans: a middle-ear comparative study
Source: Front Audiol Otol. Author manuscript; Available in PMC 2026 Sep 18. (PMC13585076; doi:10.3389/fauot.2026.1744613)
Supplement: Supplementary file 1 [file NIHMS2206123-supplement-Supplementary_Information.pdf]

Bone-Conduction Hearing in Elephants and Humans: A Middle-Ear Comparative Study

Caitlin E. O'Connell-Rodwell, Jodie L. Berezin, Anbuselvan Dharmarajan, Alessio Pignatelli,

Rachel Chen, Francisco Uzal, Xiying Guan, Sunil Puria

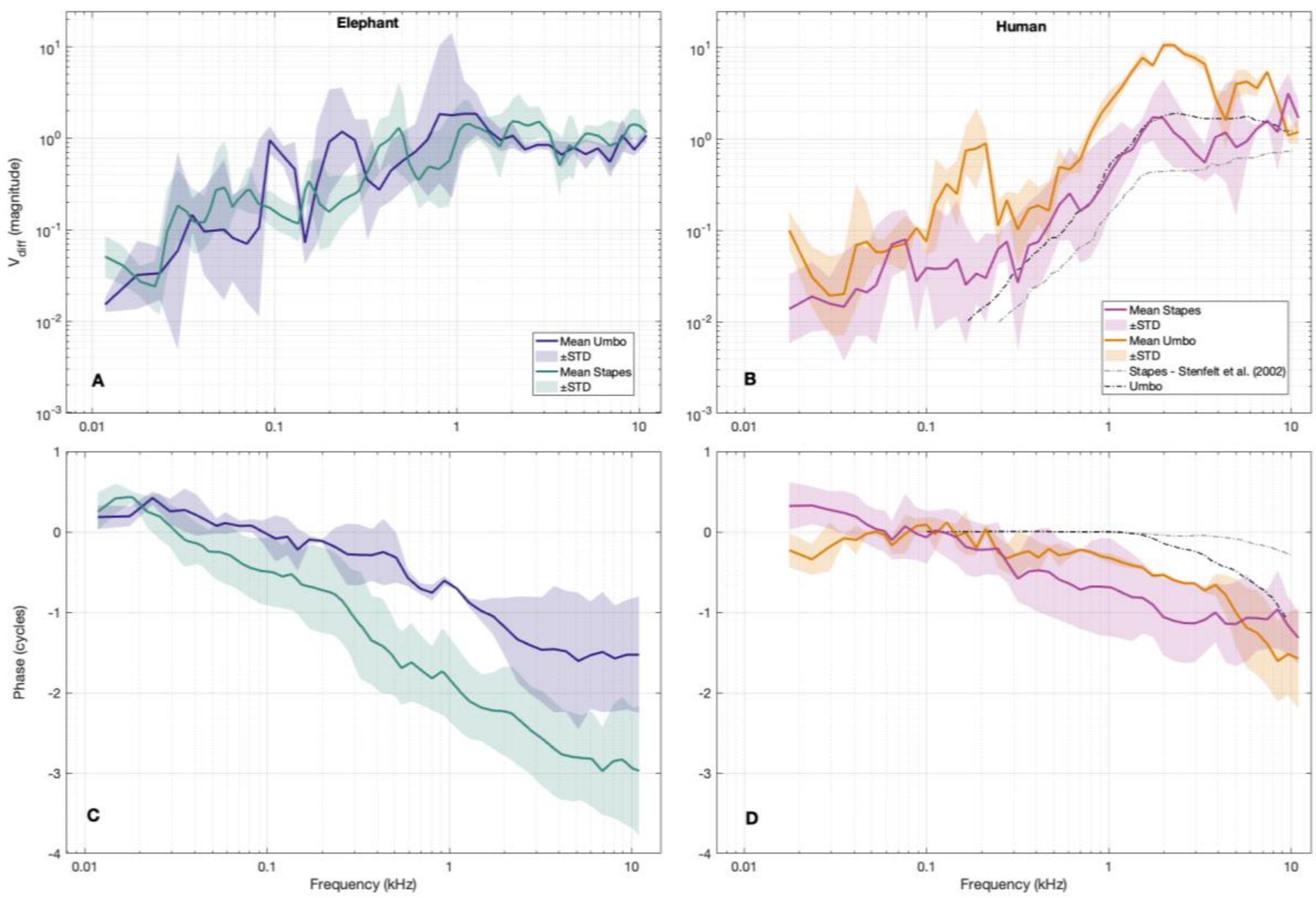

Figure S1. Same as Fig 2 but before smoothing differential motion V_{diff}. (A,C) Umbo and stapes for elephant. (B,D) Umbo and stapes for human. Top shows V_{diff} magnitude and bottom row shows phase. Also shown in panel C are the stapes and umbo V_{diff} magnitude, and in panel D the phases from V_{umbo}/V_{prom} and V_{stapes}/V_{prom} reported by Stenfelt *et al.* (2002).

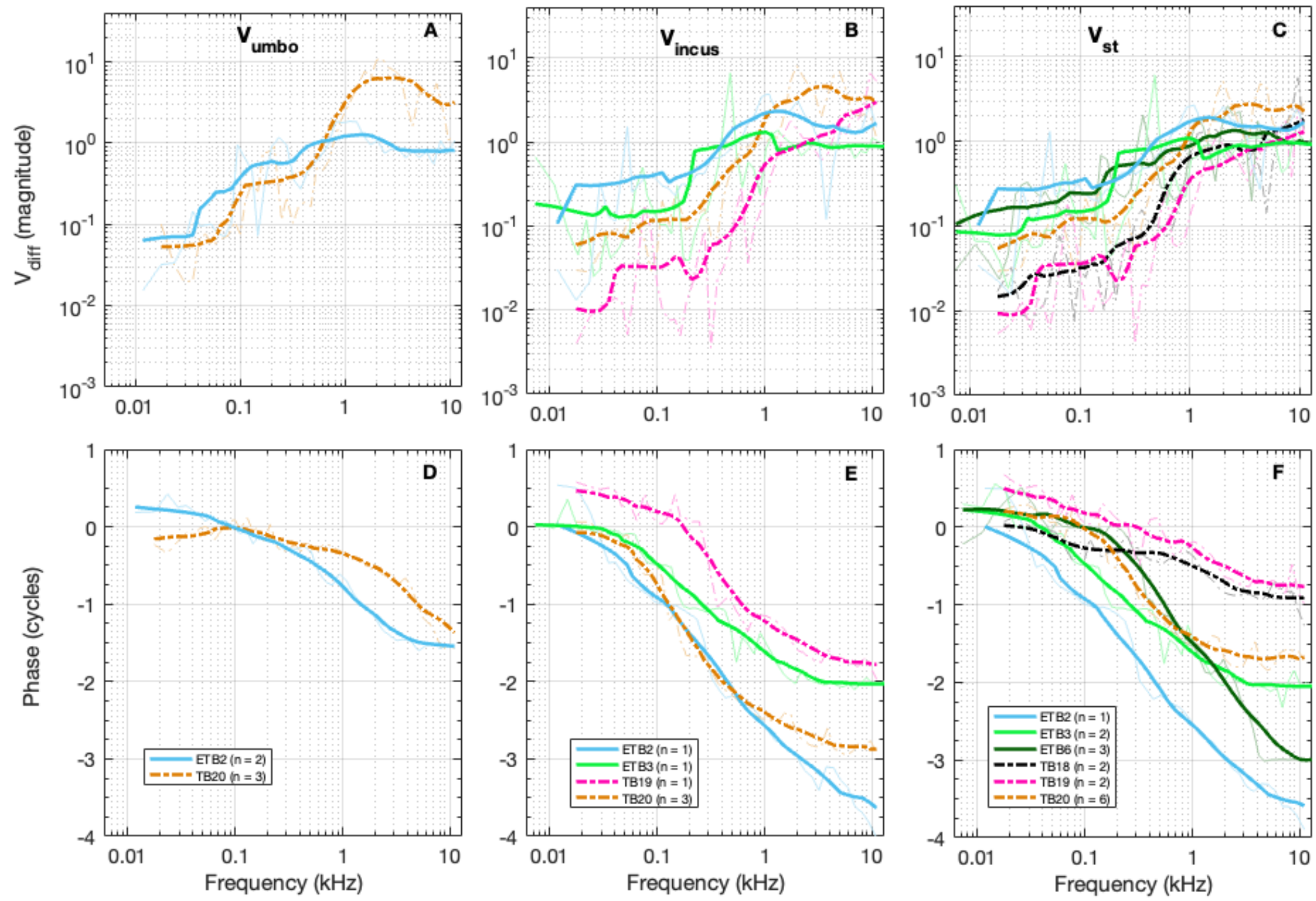

Figure S2. V_{diff} measurements from individual ears of all elephant (solid lines) and human (dashed lines) temporal bone specimens reported. The top row shows the magnitude while the bottom row shows phase. (A,D) Depicts umbo velocity. (B,E) Depicts incus (I1) velocity and phase, V_I . (C,F) Depicts the stapes velocity. A loess smoothing filter was applied to each measurement (smoothSpan=0.25).

References

Stenfelt, S., Hato, N., and Goode, R. L. (**2002**). "Factors contributing to bone conduction: the middle ear," J Acoust Soc Am **111**, 947-959.
